# Exploring Affective Priming Effect of Emotion-Label Words and Emotion-Laden Words: An Event-Related Potential Study ^†^

**DOI:** 10.3390/brainsci11050553

**Published:** 2021-04-27

**Authors:** Chenggang Wu, Juan Zhang, Zhen Yuan

**Affiliations:** 1 School of Education, Shanghai International Studies University, Shanghai 200083, China; chenggangwu@shisu.edu.cn; 2 Faculty of Education, University of Macau, E33, Avenida da Universidade, Taipa, Macau 999078, China; 3 Centre for Cognitive and Brain Sciences, University of Macau, E33, Avenida da Universidade, Taipa, Macau 999078, China; zhenyuan@um.edu.mo; 4 Faculty of Health Sciences, University of Macau, E33, Avenida da Universidade, Taipa, Macau 999078, China

**Keywords:** emotion-label words, emotion-laden words, discrete emotions, event-related potential, affective priming

## Abstract

In order to explore the affective priming effect of emotion-label words and emotion-laden words, the current study used unmasked (Experiment 1) and masked (Experiment 2) priming paradigm by including emotion-label words (e.g., sadness, anger) and emotion-laden words (e.g., death, gift) as primes and examined how the two kinds of words acted upon the processing of the target words (all emotion-laden words). Participants were instructed to decide the valence of target words, and their electroencephalogram was recorded at the same time. The behavioral and event-related potential (ERP) results showed that positive words produced a priming effect whereas negative words inhibited target word processing (Experiment 1). In Experiment 2, the inhibition effect of negative emotion-label words on emotion word recognition was found in both behavioral and ERP results, suggesting that modulation of emotion word type on emotion word processing could be observed even in the masked priming paradigm. The two experiments further supported the necessity of defining emotion words under an emotion word type perspective. The implications of the findings are proffered. Specifically, a clear understanding of emotion-label words and emotion-laden words can improve the effectiveness of emotional communications in clinical settings. Theoretically, the emotion word type perspective awaits further explorations and is still at its infancy.

## 1. Introduction

Emotion words have been widely investigated in emotion research for decades [1,2,3]. Mounting evidence has confirmed the priority of processing emotion words against neutral words [4,5,6,7,8,9]; however, these studies were cast into doubt when defining emotion words precisely [10,11,12,13,14,15,16,17,18,19,20,21,22,23,24,25].

### 1.1. Defining Emotion Words under a Distinctive Perspective

Initially, researchers in psycholinguistics and linguistics distinguished two kinds of emotion words, namely, “emotion words” and “emotion-laden words” [10,26]. Emotion words directly demonstrate affective states, such as sadness, happiness, while emotion-laden words relate to emotion without referring to certain affective states, such as death, promotion [10,26]. Although Altarriba [10] and Pavlenko [26] provided an insightful distinction between the two kinds of words, it was ambiguous to name “emotion words” as the words labeling particular affective states. There are two reasons supporting this notion. The first reason is that emotion-laden words can elicit emotion activation by the finding that emotion-laden words are processed faster than neutral words [27] and generate larger brain activation than neutral words [28]. In this sense, emotion-laden words are also emotion words. The second reason is that many previous studies [5,7,29], and even the most recent studies [9,30] on emotion words, fail to differentiate the two, making it hard to synthesize accumulating and extant evidence. Therefore, an expansive perspective is that emotion words are divided into emotion-label words and emotion-laden words. Here, emotion-label words are the same as the “emotion word” proposed by Altarriba [10] and Pavlenko [26], and emotion-laden words are unchanged [20]. In this vein, we could accommodate all of the previous efforts, regardless of whether they separated emotion-label words and emotion-laden words or not. 

A wide range of research has confirmed the distinction between emotion-label words and emotion-laden words by behavioral [15,31] and electrophysiological data [18,19,20,21,22,23,24]. The division of the two types of words is called emotion word type effect [25]. For example, one study compared emotion-label words and emotion-laden words in their priming effect and found that emotion-label words generated a larger priming effect than emotion-laden words, in both masked and unmasked conditions [14]. Kazanas and Altarriba [13] further showed the priming advantage for emotion-label words when the primes had a relatively long presentation time (i.e., 1000 ms). In addition to behavioral studies, recent event-related potential (ERP) studies also demonstrated emotion-label words elicited larger brain activation than emotion-laden words at both early [20] and late processing stages [18]. Both behavioral and electrophysiological studies supported the discrepancy between the two types of words and the superiority of emotion activation of emotion-label words over emotion-laden words. 

However, behavioral evidence for differentiating the two kinds of emotion words is not conclusive [16,32,33]. For example, Martin and Altarriba [16] found emotion-label words and emotion-laden words were recognized similarly in the lexical decision task. Vinson et al. [33] also reported that the emotion word type was not able to influence lexical processing. Therefore, Hinojosa et al. [32] claimed that more research is needed to tap into the distinction between the two kinds of emotion words. 

Based on the debate of whether emotion-label words and emotion-laden words are distinct, the current study aimed to further explore the distinctiveness of the two kinds of words in a new paradigm that is affective priming paradigm and will be introduced in the next section. 

### 1.2. Affective Priming in Emotion Words

Affective priming is a modified version of the priming paradigm and has been explored for several decades [34,35,36]. In an affective priming study, the researchers explicitly manipulate the valence relationship between the prime words and target words, and the primes and targets can be different or the same in valence (negative or positive). There is a strong affective priming effect that a target word (e.g., happy) is facilitated by a congruent word that shares the same valence with the target word (e.g., kind), compared to the target word that is preceded by an incongruent word that has the different valence from the target word (e.g., regret). More crucially, the affective priming effect can be even obtained in the masked condition that the participants were unaware of the primes [37]. From then on, the affective priming effect has been examined by various emotion stimuli, including affective pictures [38], emotional facial expressions [39], and emotion words [36,40,41,42,43,44]. 

For example, Altarriba and Canary [41] examined how emotion-laden words primed emotion-laden words in the affective priming paradigm by manipulating the arousal of primes. The results clearly revealed that high arousal and moderate arousal emotion-laden words produced equal affective priming effects (63 ms and 62 ms). Yao and Wang [36] recently provided electrophysiological evidence of affective priming effects in Chinese. The results showed that late positive component (LPC) was larger in the affective incongruent condition than in the affective congruent condition. LPC is an event-related potential (ERP) component that peaks at around 500 ms after the onset of the stimuli and is associated with emotion stimuli evaluation [2]. Enhanced LPC in incongruent condition suggests it is more effortful to evaluate the valence of the targets if the targets and primes are different in valence than when the targets and primes are the same in valence. Yao and Wang [36] further showed that the reduction of LPC induced by targets that are preceded by congruent primes was restricted within abstract positive words (e.g., talent). No differences between congruent and incongruent trials were found for concrete positive words (e.g., money), suggesting that the affective priming effect is modulated by concreteness. 

It is clear that using the affective priming paradigm to examine the processing characteristics of emotion words has been firmly established, but the studies of emotion word processing that used the affective priming paradigm did not attend to the division of the two types of emotion words. Therefore, the current study followed the prior studies to explore affective priming of emotion-label words and emotion-laden words. 

### 1.3. The Present Study

Although there are many studies using the affective priming paradigm to explore emotion word processing, emotion word type has not received empirical attention. The present study aimed to examine how emotion word type influences the affective priming effect by measuring behavioral performance and brain electrophysiological activation. Two experiments using unmasked (Experiment 1) and masked (Experiment 2) paradigms were conducted to compare the affective priming effect of emotion-laden words and -label words as primes. The major differences between unmasked and masked paradigms are that the primes are presented longer (e.g., 100–1000 ms) in unmasked priming paradigm than in masked priming (e.g., 50–100 ms), and there are usually a forward and a backward mask presented before and after the primes, making participants unaware of the primes. In the two priming experiments, emotion-laden words were always the targets, which enabled us to directly compare the priming of emotion-label words and emotion-laden words, namely, how the two types of words influenced the same targets (all emotion-laden words) processing. 

The current study focused on two ERP components. The first one is LPC. As mentioned above, LPC was an emotion word recognition-related ERP component. For example, emotion-laden words evoked increased LPC than emotion-label words in a second language, suggesting that LPC was susceptible to emotion word type [18]. LPC is also sensitive to valence, with increased LPC being elicited by the positive words than negative words [45]. However, there was also contradictory evidence showing that positive words provoked decreased LPC than negative words, suggesting there were other factors influencing the amplitude of LPC [2]. It was also found that LPC was easily identified in lexico-semantic tasks, such as valence judgment tasks and lexical decision tasks. Since the current study attempted to use the valence judgment task, LPC was selected. We predicted that LPC would be elicited increasingly by emotion-laden words than by emotion-label words. As for valence, based on the previous studies [20,46], it is predicted that negative words elicited greater LPC than positive words. Another factor that congruency is also assumed to be able to influence LPC amplitude. Precisely, larger LPC is predicted to be elicited by incongruent trials than by congruent trials because processing incongruent trials is more difficult than congruent trials. 

In addition to LPC, early posterior negativity (EPN) is also an emotion-processing-related early ERP component with an occipital distribution, and emotion words often elicit larger EPN than neutral words [2]. EPN has been robustly found to reflect the automatic and fast emotion activation of the emotion words, and the enhancement of EPN elicited by emotion words against neutral words has been observed in many tasks [2]. Therefore, it is hypothesized that emotion-laden words, primed by emotion-label words, would elicit larger EPN than those primed by emotion-laden words due to the notion that emotion-label words could engender greater emotion activation than laden words. However, since EPN is a relatively early ERP marker and related to automatic emotion activation, valence and congruency are assumed to be irrelevant to EPN.

## 2. Experiment 1

The experiment was designed to explore the affective priming effect of emotion-label words and emotion-laden words in an unmasked priming paradigm, that is, emotion-label words and emotion-laden words as primes were not masked and presented at a relatively short time (i.e., 250 ms). 

### 2.1. Method

#### 2.1.1. Participants 

A total of 22 Chinese speakers from the University of Macau were recruited for the current experiment. The recruitment was achieved through the notification in various WeChat groups that included hundreds of students from various faculties. Interested participants would contact the experimenter and determine the time slots of the experiments. All of the participants were college students from the University of Macau. They were paid MOP 100 (around USD 13) per hour for participating. Chinese is their first language, and their second language is English. They voluntarily participated in Experiment 1 and signed the consent forms before the experiment. Two of them were excluded due to their extensive artifacts during the recording. Therefore, 20 (5 males, mean age: 23.42 years old) participants were retained for the analysis. Gender distribution was not even. It was possible that female students were more interested in language experiments than male students. None of them were with psychiatric disorders and all of them had normal and corrected-to-normal vision with right-handedness. The sample size was determined by the G*power [47]. Provided that effect size was medium (partial *η*^2^ = 0.1) and power is 0.80, 20 participants were required, namely, critical *F* (1,19) = 4.38, *p* = 0.05, also following previous studies [21]. 

#### 2.1.2. Materials 

Overall, 40 negative emotion-label words, 40 positive emotion-label words, 40 negative emotion-laden words, and 40 positive emotion-laden words that were matched on strokes, word frequency, and arousal (*Fs* < 1) are used as primes [23]. A group of participants who did not involve in the present experiments were asked to rate the valence (negativity and positivity) and arousal of the four kinds of emotion words. Arousal is the extent to which the emotion is induced by the emotion words. The negative words were rated to be more negative than positive words, *F* (3,156) = 473.454, *p* < 0.001, while no difference was found for emotion-label words and emotion-laden words, *ps* > 0.05 (see Table 1 for more information). As for target words, another 80 negative emotion-laden words and 80 positive emotion-laden words that were divided into 40 words in each category [48] were selected. The four groups of words were matched on word frequency, strokes, and arousal, *ps* > 0.2 (see Table 2 for more details). Word frequency was retrieved from SUBTLEX-CH [49], whereas the arousal data was obtained from Yao et al. [46]. The target words were randomly allocated to the four priming types (negative emotion-label words, positive emotion-label words, negative emotion-laden words, and positive emotion-laden words). Valence was also confirmed, *F* (3,156) = 141.265, *p* < 0.001; however, no difference was found between the words primed by the emotion-label words and emotion-laden words, respectively, *ps* > 0.05.

#### 2.1.3. Procedure

**Figure 1:**
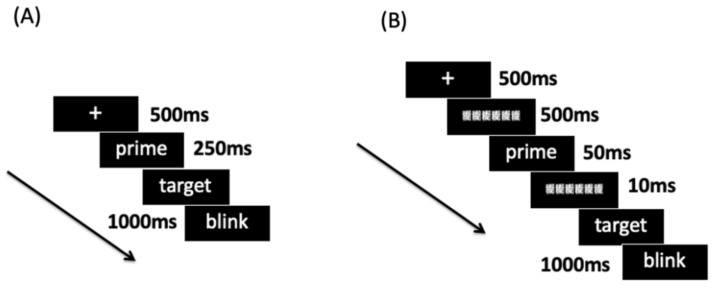
Trial schemes for Experiment 1 (**A**) and Experiment 2 (**B**).

The participants were seated at a distance of 70 cm from the computer screen in a dim light chamber. The experimenter first explained the procedure and the task requirement that instructed the participants to decide whether the target words were positive or negative. In Experiment 1, for one test trial, before the target, a fixation (500 ms) and a prime (250 ms) were presented to the participants in sequence. After the presentation of a prime, a target word would be displayed on the screen to suggest the participants should decide the valence of the target word. In all trials, the primes could be congruent (i.e., in the same valence) or incongruent (i.e., in the different valence) with the target words. In this way, target words could be followed by the primes whose valence was incongruent or congruent with the target words (see Figure 1). The target word disappeared as soon as the participants made a response. Between the trials, a 1000 ms blink window suggested that participants could blink their eyes. Altogether, there are 320 trials collapsed into 8 blocks with 40 trials in one block. The blocks and trials in one block were randomly sequenced. Between the blocks, participants were allowed to have a short rest. 

#### 2.1.4. ERP Recording and Analysis 

The electroencephalography (EEG) data were collected with a 129-electrode HydroCel Geodesic Sensor Net (Electrical Geodesics, Inc., Eugene, OR, USA). Impendence during the recording was retained under 50 kΩ. The sampling rate was 1000 Hz, and the online bandpass filter was between 0.1–30 Hz. The Net Station Waveform Tools were used to analyze the offline data. The data were firstly filtered with a high-frequency pass of 0.1 Hz and a low-frequency pass of 30 Hz. The filtered EEG data were segmented into an 750 ms epoch surrounding the trigger with 100 ms before the stimuli onset as a baseline. Following segmentation, artificial detection was performed to discard the epochs that are contaminated by extreme amplitude differences during the whole recording (bad channel; ±200 μV), vertical electrooculogram (eye blinks; ±70 μV) and horizontal electrooculogram (eye movements; ±27.5 μV). Trials with more than 10% artifact violations in channels were eliminated. The segments were corrected with a 100 ms baseline prior to the trigger. The EEG data are referenced to the average of all the electrodes. For EPN, six electrodes were selected, following previous studies [18,20], namely, P7/P8, P9/P10, and PO7/PO8, within the time window of 190 ms to 350 ms. For LPC, three right central sites were selected (C2, C4, and C6) within the time window of 400 ms to 680 ms. 

### 2.2. Results

#### 2.2.1. Behavioral Results 

Data removal was applied for the trials that exceeded M ± 2.5 SD, thereby excluding 1.73% trials. A 2 (word type: emotion-label words and emotion-laden words) × 2 (valence: negative and positive) × 2 (congruency: incongruent and congruent) repeated-measure ANOVA was conducted for accuracy rate and reaction time. The three factors were all within-subject factors. 

For accuracy rate, a main effect of word type was observed, *F* (1, 19) = 7.694, *p* < 0.05, partial *η*^2^ = 0.288. Emotion-laden words (0.974) had higher accuracy rate than emotion-label words (0.960) as primes. Positive words (0.977) were processed more accurately than negative words (0.957), *F* (1, 19) = 6.123, *p* < 0.05, partial *η*^2^ = 0.244. Moreover, there was an interaction between word type and valence, *F* (1, 19) = 5.449, *p* < 0.05, partial *η*^2^ = 0.223. Post hoc comparisons revealed that accuracy rate for negative emotion-laden words as primes (0.967) was higher than that for negative emotion-label words as primes (0.946), *t* (19) = 3.384, *p* < 0.01. Positive emotion-laden words (0.980) and emotion-label (0.974) words had similar accuracy rates *t* (19) = 1.037, *p* > 0.1. No other main effect or interactions was found, *ps* > 0.05. 

As for reaction time, we only identified an interaction between valence and congruency *F* (1, 19) = 5.290, *p* < 0.05, partial *η*^2^ = 0.218. Post hoc comparisons showed that in congruent condition, negative words (975.96 ms) had higher reaction time than in incongruent condition (933.37 ms), *t* (19) = 2.770, *p* < 0.05, while no difference was found for positive words between congruent (924.34 ms) and incongruent conditions (947.68 ms), *t* (19) = 0.942, *p* > 0.1 (see Table 3 for more details). 

#### 2.2.2. ERP Results

##### EPN

**Figure 2:**
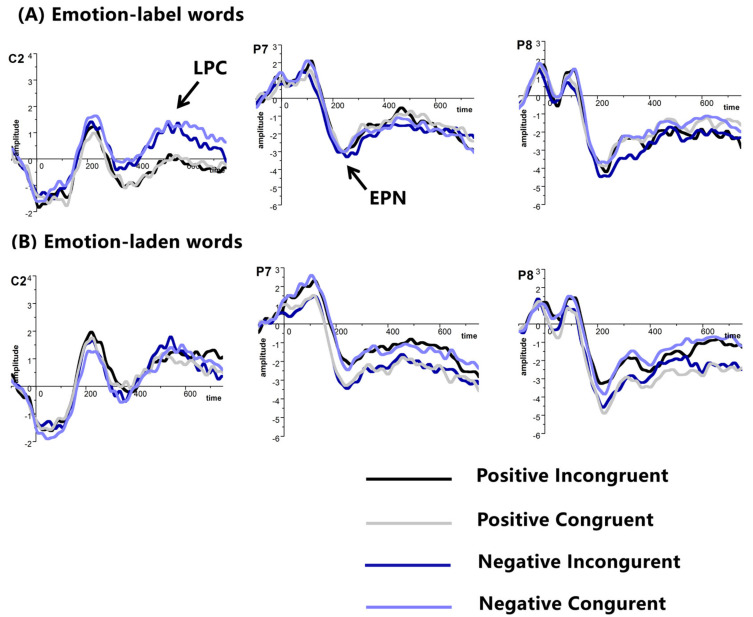
Grand average ERPs of EPN and LPC at selected electrodes with (**A**) emotion-label words, and (**B**) emotion-laden words as primes in Experiment 1, amplitude (μV), time (ms).

A 2 (word type: emotion-label words and emotion-laden words) × 2 (valence: negative and positive) × 2 (congruency: incongruent and congruent) × 3 (electrodes) × 2 (hemisphere: left and right) repeated-measure ANOVA was performed. Negative words (–3.495 μV) evoked larger EPN than positive words (−3.180 μV), *F* (1, 19) = 4.431, *p* < 0.05, partial *η*^2^ = 0.189. We observed the main effect of electrode, *F* (2, 38) = 7.883, *p* < 0.01, partial *η*^2^ = 0.293, with ERP that was elicited at P7/P8 (−2.743 μV) being smaller than that at P9/P10 (−3.913 μV) and PO7/PO8 (−3.357 μV). There was an interaction between valence and congruency, *F* (1, 19) = 10.452, *p* < 0.01, partial *η*^2^ = 0.355. Positive words elicited larger EPN in congruent condition (–3.433μV) than in incongruent condition (−2.927 μV), whereas negative words provoked smaller EPN in congruent condition (−3.090 μV) than in incongruent condition (−3.900 μV). No other main effect or interaction was identified, *ps* > 0.05 (see Figure 2).

##### LPC

A 2 (word type: emotion-label words and emotion-laden words) × 2 (valence: negative and positive) × 2 (congruency: incongruent and congruent) × 3 (electrodes) repeated-measure ANOVA was performed. The main effect of word type was observed, *F* (1, 19) = 5.809, *p* < 0.05, partial *η*^2^ = 0.234, such that larger LPC was elicited by emotion-laden words that were primed by emotion-laden words (0.556 μV) than those primed by emotion-label words (0.033 μV). No other main effect or interaction was found. 

### 2.3. Discussion

The result confirmed the processing advantage of negative emotion-laden words over emotion-label words in valence evaluation [19]. In one recent study, we found that negative emotion-laden words were easier to decide if the word was negative or positive than negative emotion-label words [19]. For accuracy rate, only negative emotion-laden words had a higher accuracy rate than negative emotion-label words, suggesting that in valence evaluation, discrete negative emotions as indicated by emotion-label words made it hard to merge a single dimension, which impaired the performance [19]. However, negative emotion-laden words connect to the emotion-label words in unpredictable ways. For example, death can activate one’s sadness, worry, and anxiety, etc. It is possible that negative emotion-laden words’ multiple connections facilitate valence evaluation. In contrast to negative words, positive emotion-label and emotion-laden words had similar accuracy rate, suggesting that positive emotions are merged in density. 

Negative emotion-label words actually inhibited the target negative emotion-laden words, while no priming effect was found for positive words behaviorally, consistent with the notion that emotions are discrete rather than valence based. As for ERP data, in contrast with our hypothesis, we found an inhibition effect for negative words and a facilitation effect for positive words in EPN, with enhanced EPN that was elicited in congruent condition than in incongruent condition for positive words, whereas decreased EPN was evoked in congruent condition than in incongruent condition for negative words. The results of LPC were in agreement with the findings of one previous study showing that emotion-laden words generated larger LPC than emotion-label words in a lexical decision task [18]. 

EPN that was elicited at P9/P10 and PO7/PO8 was larger than the EPN at P7/P8. The differences between the amplitude of EPN were not novel. It was found that early negativity occurring at P9/P10 was usually larger than other sites, such as P7/P8 [20]. 

## 3. Experiment 2

Experiment 2 was aimed at extending the findings of Experiment 1 by adding a mask for the primes (500 ms) and shortening the presentation time (50 ms). Due to the limited exposure of the primes to the participants, it would allow us to explore whether automatic activation of the primes could influence the emotion-laden target words in the same way or not.

### 3.1. Method

#### 3.1.1. Participants 

A total of 31 participants (mean age: 22.45 years old, two males) from the same population of Experiment 1 were recruited for the present experiment after excluding four participants who produced extensive artifacts. All of the participants were right-handed and without psychiatric disorders. They also had a normal or corrected-to-normal vision. None of them participated the Experiment 1. 

#### 3.1.2. Materials

The stimuli were identical to Experiment 1. 

#### 3.1.3. Procedure 

The whole procedure was the same as Experiment 1, except that the primes were presented for 50 ms and were masked by a forward mask for 500 ms and a backward mask for 10 ms (see Figure 1). The mask was constructed by overlapping several complex Chinese characters.

#### 3.1.4. ERP Recording and Analysis

The EEG recording was the same as Experiment 1. For data analysis, EPN and LPC were identified and electrode selection was in line with Experiment 1, but the time windows were changed due to the paradigm changes, namely, EPN (170–310 ms) and LPC (370–690 ms). 

### 3.2. Results 

#### 3.2.1. Behavioral Results

Trials that exceeded 2.5 SD ± Mean were deleted for further analysis, thereby discarding 3.03% data. The means of reaction time and accuracy rate are displayed in Table 4. For accuracy rate, emotion-laden target words that were preceded by emotion-label words (0.943) were recognized less accurate than those preceded by emotion-laden words (0.952), *F* (1, 30) = 4.976, *p* < 0.05, partial *η*^2^ = 0.142. Further comparisons showed that positive target words preceded by negative emotion-label words had a lower accuracy rate than those preceded by negative emotion-laden words, *t* (30) = 2.449, *p* < 0.05, while no differences between emotion-label words and emotion-laden words were found for other comparisons, *ps* > 0.1. Positive words (808.33 ms) were also evaluated faster than negative words (833.81 ms), *F* (1, 30) = 5.103, *p* < 0.05, partial *η*^2^ = 0.145. No other main effect or interaction reached significance. 

#### 3.2.2. ERP Results

##### EPN

**Figure 3:**
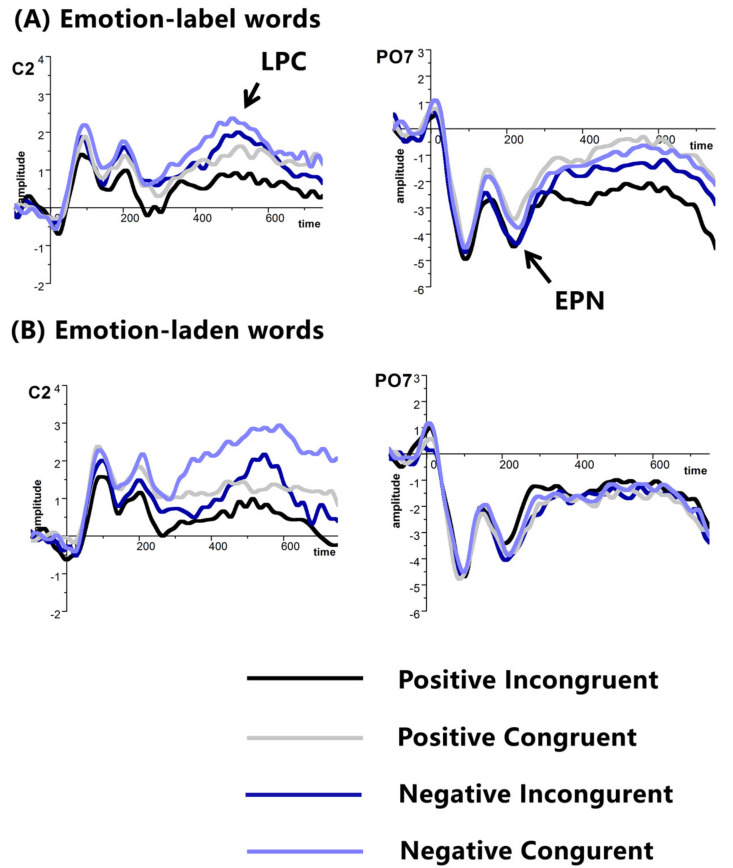
Grand average ERPs of EPN and LPC at selected electrodes with (**A**) emotion-label words, and (**B**) emotion-laden words as primes in Experiment 2, amplitude (μV), time (ms).

The main effect of electrode was observed, *F* (2, 60) = 21.525, *p* < 0.001, partial *η*^2^ = 0.418. The amplitude of EPN was larger at P9/P10 (−4.499 μV) than P7/P8 (−2.765 μV) and PO7/Po8 (−2.953 μV). We also found an interaction among word type, valence, congruency, electrode, and hemisphere, *F* (2, 60) = 5.074, *p* < 0.05, partial *η*^2^ = 0.145. Post hoc comparison confirmed that only positive emotion-laden words that were primed by negative emotion-label words (−3.482 μV) elicited larger EPN than positive emotion-laden words primed by negative emotion-laden words (−2.340 μV), *t* (30) = 2.315, *p* < 0.05 at PO7. No other main effect or interaction was found (see Figure 3). 

##### LPC

The main effect of electrode was identified, *F* (2,60) = 9.079, *p* < 0.001, partial *η*^2^ = 0.232. Pairwise comparisons showed that smaller LPC was evoked at C6 (0.311μV) than that at C4 (0.786 μV) and C2 (1.362 μV). We also found the main effect of valence, *F* (1,30) = 8.719, *p* < 0.01, partial *η*^2^ = 0.225, such that negative words (1.157 μV) evoked larger LPC than positive words (0.482 μV). No other main effect or interaction was observed. 

### 3.3. Discussion 

The behavioral data from the current experiment only confirmed the advantage of positive word processing over negative words, in line with many previous studies showing the same advantage for positive words [13,14]. 

It is worth noting that emotion-label words as primes inhibited the target emotion words processing, a finding similar to the one in Experiment 1. Further comparisons between the two types of words revealed that only under incongruent conditions, negative emotion-label words inhibited positive word processing against negative emotion-laden words. ERP results further showed that negative emotion-label words inhibited positive word recognition that elicited increased EPN than that was provoked by positive words preceded by negative emotion-laden words. The combined ERP and behavioral findings suggested that emotion word type could still modulate emotion word recognition in masked priming paradigm, at least for negative ones. 

In addition, negative words generated enhanced LPC than positive words, in agreement with many previous studies [20,46]. These ERP findings were additionally supported by the reaction time data showing that negative words were recognized slower than positive words [13,14].

The largest EPN was elicited at P9/P10 among other sites such as P7/P8 and PO7/PO8. This finding was in agreement with the result of Experiment 1. It is possible that early brain activation during emotion word processing was more noticeable at P9/P10 than other occipital-temporal sites. A larger LPC was generated at C2 than C4 and C6, suggesting the closer it is to the middle of the brain, the more salient LPC is. 

## 4. General Discussion

The primary goal of the present study was to investigate how emotion-laden words and emotion-label words influence emotion-laden target words in unmasked (Experiment 1) and masked (Experiment 2) affective priming paradigms. The present Experiment 1 showed that positive words produced a priming effect, while negative words generated an inhibition effect, suggesting that the affective priming effect was restricted within positive words. In addition, the distinction between emotion-label words and emotion-laden words was found to modulate emotion word processing explicitly (Experiment 1) and implicitly (Experiment 2). 

In a typical affective priming paradigm, a prime that could be an emotion-label word and an emotion-laden word is shortly presented (250 ms or 50 ms), and participants are required to decide the valence of the target emotion-laden words that could be congruent (in the same valence) or incongruent (in the opposite valence) with the primes. Previous studies that did not separate the two kinds of words have identified the affective priming effect that target words were facilitated when the primes and targets are in the same valence [35]. For example, one recent study orthogonally manipulated concreteness of the words and semantic relatedness between the primes and targets in the affective priming paradigm. The results found the overall affective priming effect for both concrete and abstract words [50]. However, the affective priming effect was not as genuine as Ferre and Sanchez-Casas [49] assumed. Actually, there was a strong interaction among valence, semantic relatedness, and affective congruency. The planned comparisons showed that there was an affective priming effect for positive words in semantically unrelated pairs, whereas for negative words, the affective priming effect was identified in semantically related pairs. These results indicated that the affective priming effect in words was complex and modulated by valence and semantic relatedness. Similarly, Padovan et al. [51] did not find affective priming effect for negative primes and targets (e.g., spy–famine) that do not have semantic overlap. Rather, emotionally congruent negative primes inhibited target negative word processing (947 ms) against unrelated prime and target pairs (e.g., journal–famine, 921 ms). This pattern of results suggests that, at least for negative words, when primes and targets are unrelated in semantics, the affective priming effect was not that reliable. 

Apart from valence, concreteness, and semantic relatedness, one issue that has not been addressed in previous studies is emotion word type. The present study distinguished emotion-laden words (e.g., butterfly, mosquito) and emotion-label words (e.g., joy, fear) and compared the affective priming effect of the two kinds of words on emotion-laden target words. In Experiment 1, the primes were not masked; therefore, the participants were aware of the primes. The results showed inhibition of negative prime words, such that negative target words were processed slower when primed by the negative words (congruent condition) than primed by the positive words (incongruent condition). Moreover, enhanced EPN was elicited by the negative words in an incongruent condition than in a congruent condition. These findings were in line with the study of Padovan et al. [50] that negative words as primes actually inhibited the target negative words. However, it was noted that word stimuli in the study of Padovan et al. [50] were probably mixed with emotion-label words and emotion-laden words or included only emotion-laden words (e.g., spy, famine). The current study contained both types of words and found similar results, indicating the inhibition effect of negative prime words in the affective priming paradigm could be found in both emotion-laden words and emotion-label words. 

As for positive words, although behavioral results did not show the differences between congruent trials and incongruent trials, ERP results indicated that positive words actually produced an affective priming effect. Specifically, increased EPN was provoked by the positive words that were preceded by the positive words than the positive words preceded by negative words. This finding suggested that positive words are merged in valence, and positive emotions are more similar to each other than negative emotions [52], allowing the positive words to generate an affective priming effect. 

However, we did not find the interaction between congruency and emotion word type, indicating that the affective priming effect seems to be irrelevant to emotion word type. For example, emotion-laden words that were primed by emotion-laden words induced larger LPC than those primed by emotion-label words; however, congruency failed to influence LPC. These results demonstrated that emotion-label words facilitated emotion-laden target word processing [18], at least in unmasked conditions, and this facilitating effect was unrelated to the affective priming effect. It is plausible that the differences between the two types of words are attributed to emotion activation, and this activation was equal in both congruent and incongruent trials. 

In Experiment 2, when primes were masked and presented briefly, the affective priming effect was hard to identify. However, it was different for the influence of primes of emotion-label words and emotion-laden words on emotion-laden target words. Emotion-laden target words preceded by emotion-label words were more difficult to evaluate the valence than those preceded by emotion-laden words. Electrophysiological evidence further revealed that positive emotion-laden words preceded by negative emotion-label words elicited larger EPN than those preceded by negative emotion-laden words. This finding demonstrated that enhanced emotion activation induced by emotion-label words, at least for negative words, could further influence emotion-laden word processing at the neural level, in line with previous examinations [22,23,24].

Another lexical variable that has not been controlled prior to the experiment is the word association between the primes and targets since the word association was found to influence the affective priming effect [43]. We have retrieved the word association between the primes and targets in four conditions (emotion-label words incongruent: 0.011; emotion-label words congruent: 0.002; emotion-laden words incongruent: 0.011; emotion-laden words congruent: 0.027; and two words were not found in emotion-laden word congruent condition) from one recent Chinese Lexical Association Database [53]. There was extremely low word association in the four conditions. Further ANOVA tests showed the word association was negligibly low across four conditions, emotion word type: *F* (2,77) = 1.683, *p* > 0.20, congruency: *F* (2,77) = 2.391, *p* > 0.13, interaction between the two factors: *F* (2,77) = 0.237, *p* > 0.60. Based on the word association data, it is argued that there is no semantic association between primes and targets in both incongruent and congruent conditions. Therefore, the restrained affective priming effect in the present study was attributed to the low word association between the primes and targets. For example, Hu and Liu [43] recently found that when the word association strength was low for the primes and targets, there was no affective priming effect, in agreement with the current findings. It is possible that in order to identify reliable and significant affective priming effect, relatively high word association between the primes and targets is obligatory. Future studies could manipulate word association strength between emotion-label words and emotion-laden words and explore the relationship between the association strength and affective priming effect. 

Some limitations of the present study should be noted and some questions could be explored in future studies. As mentioned before, we did not manipulate the word association between primes and targets. Therefore, it remains unclear how the word association strength influences the affective priming of emotion-laden words and emotion-label words. Additionally, it could be argued that other neuroimaging methods (e.g., functional near-infrared spectroscopy, fNIRS) with enhanced spatial resolution can be used to further explore the affective priming of emotion-label words and emotion-laden words. Another limitation was that the participants were dominant in female students, suggesting a possibility of gender bias toward emotion word processing. Although there is evidence showing that female and male participants rated emotion words similarly on context availability, emotionality, concreteness, and imageability [54], it is still worth further exploration on gender effect on the processing of emotion-label words and emotion-laden words. Finally, as one reviewer pointed out, the prestimulus baseline in Experiment 1 was relatively active. It is probably a result of the unmasked affective priming paradigm. The primes were not masked and presented for 250 ms. This operation might naturally make the influence of primes visible and durative, and therefore, the noise present in the baseline was the lasting influence of the primes. However, Experiment 2 adopting the masked priming paradigm showed very small noise in the baseline. This demonstrated the essence of combining the two experiments to reach reliable conclusions. 

The current study, along with the previous studies on emotion and language [14], has the clinical implication that since it is important for the clients and therapists to correctly understand and express emotions with language, the crucial role of emotion-label words and emotion-laden words in emotional communication is straightforward. Specifically, emotion-label words, since they are closer to emotional states, are of utmost importance to convey and comprehend emotions. Therefore, learning and training in the acquisition of emotion-label words are helpful to improve the effectiveness of emotional communication in clinical settings. However, it is also worth emphasizing that emotion-laden words (e.g., war, disaster) can help to establish contexts to learn the emotion-label words. In this sense, only by combining the two kinds of emotion words can we construct a holistic emotional life. Theoretically, the present study followed the conceptual framework of emotion-label words and emotion-laden words—an emotion word type perspective on emotion words. This perspective was still at its infancy and open to debate and critique [25,32], thereby creating a new arena for further explorations of emotion words. 

## 5. Conclusions

The present study examined how primes of emotion-label words and emotion-laden words influenced emotion-laden target words in masked and unmasked affective priming paradigms. The results disentangled emotion-label words from emotion-laden words by showing that emotion-laden words primed by emotion-label words were processed less accurately in both experiments and elicited larger LPC than those primed by emotion-laden words. The discrepancy between the two kinds of words was further validated in the findings that negative emotion-label words led positive target words to generate enhanced EPN than those preceded by the negative emotion-laden words in the masked affective priming paradigm. These findings underscored it is obligatory to define emotion words under an emotion word type perspective [25]. 

## Figures and Tables

**Table 1:** Mean and standard deviation (SD) in brackets for word characteristics for Chinese emotion-label words and emotion-laden words as primes.

	Emotion-Label Words		Emotion-Laden Words	
	Negative	Positive	Negative	Positive
Sample	心疼 (sadness)	舒心 (joy)	分手 (breakup)	生日 (birthday)
Word frequency	2.37 (0.86)	2.22 (0.84)	2.30 (0.67)	2.37 (0.63)
Strokes	16.78 (4.24)	17.80 (3.31)	16.23 (4.31)	16.55 (5.04)
Arousal	4.78 (0.29)	4.78 (0.47)	4.75 (0.45)	4.88 (0.67)
Valence	2.58 (0.32)	5.29 (0.48)	2.32 (0.57)	5.34 (0.51)

**Table 2:** Mean and standard deviation (SD) in brackets for word characteristics for Chinese emotion-laden words as targets.

	Emotion-Laden Words Primed by Label Words		Emotion-Laden Words Primed by Laden Words	
	Negative	Positive	Negative	Positive
Sample	死囚 (prisoner)	花束 (flower)	火化 (cremation)	春光 (spring view)
Word frequency	1.98 (0.79)	1.97 (0.69)	2.14 (0.64)	1.82 (0.75)
Strokes	17.43 (3.94)	18.08 (4.31)	17.70 (4.82)	16.78 (4.86)
Arousal	5.58 (1.36)	5.52 (1.03)	5.43 (1.03)	5.40 (0.91)
Valence	3.49 (1.07)	5.91 (0.63)	3.17 (0.84)	5.98 (0.60)

**Table 3:** Mean reaction time (ms) and accuracy rate (%) in brackets of emotion-label words and emotion-laden words as a function of congruency and valence (Experiment 1).

	Negative		Positive	
	Congruent	Incongruent	Congruent	Incongruent
Emotion-label	983.05 (94.05)	926.79 (95.20)	908.24 (97.58)	936.31 (97.32)
Emotion-laden	968.88 (96.31)	939.95 (97.18)	940.43 (98.21)	959.05 (97.83)

**Table 4:** Mean reaction time (ms) and accuracy rate (%) in brackets of emotion-label words and emotion-laden words as a function of congruency and valence (Experiment 2).

	Negative		Positive	
	Congruent	Incongruent	Congruent	Incongruent
Emotion-label	832.16 (95.15)	838.15 (93.51)	792.99 (94.63)	812.03 (93.74)
Emotion-laden	830.51 (95.59)	834.43 (95.54)	806.23 (94.93)	814.09 (94.93)

## Data Availability

The data are available from the corresponding author upon reasonable requests.
